# Mild behavioral impairment in idiopathic REM sleep behavior disorder and Lewy body disease continuum

**DOI:** 10.1007/s00702-024-02877-w

**Published:** 2025-01-09

**Authors:** Bora Jin, Eun Jin Yoon, Kyung Ah Woo, Seoyeon Kim, Seungmin Lee, Ryul Kim, Jung Hwan Shin, Yu Kyeong Kim, Jee-Young Lee

**Affiliations:** 1 Department of Neurology, Seoul Metropolitan Government-Seoul National University Boramae Medical Center, Seoul National University of College of Medicine, Seoul, Republic of Korea; 2 Department of Nuclear Medicine, Seoul Metropolitan Government-Seoul National University Boramae Medical Center, Seoul National University of College of Medicine, Seoul, Republic of Korea; 3 Department of Neurology, Seoul National University Hospital, Seoul National University College of Medicine, Seoul, Republic of Korea; 4 Neuroscience Research Institute, Medical Research Center, Seoul National University, Seoul, Republic of Korea

**Keywords:** Mild behavioral impairment, Idiopathic REM sleep behavior disorder, Parkinson’s disease, Dementia with Lewy bodies, Lewy body disease

## Abstract

To investigate the clinical impact of mild behavioral impairment (MBI) in a predefined cohort with Lewy body disease (LBD) continuum. Eighty-four patients in the LBD continuum participated in this study, including 35 patients with video-polysomnography-confirmed idiopathic REM sleep behavior disorder (iRBD) and 49 clinically established LBD. Evaluations included the Movement Disorder Society-Unified Parkinson’s Disease Rating Scale (MDS-UPDRS), neuropsychological tests, and MBI Checklist (MBI-C). We examined the clinical associates of MBI-C and its diagnostic values in predicting disease severity and cognitive impairment across the LBD continuum. Participants were classified into 19 cognitively normal (CN), 45 mild cognitive impairment (MCI), and 20 dementia groups. Median MBI-C total scores were 1.0, 8.0, and 18.5 for CN, MCI, and dementia groups, respectively, with a significant increasing trend (p < 0.001). The MBI-C total score demonstrated significant correlations with the MDS-UPDRS part 1 (r = 0.504, p < 0.001) and total scores (r = 0.508, p < 0.001). Furthermore, significant correlations were observed between MBI-C and cognitive performances in frontal/executive (DSC: r = −0.314, p = 0.006; TMT-B: r = −0.338, p = 0.003) and attentional (TMT-A: r = −0.343, p = 0.002) domains. A cutoff 5.0 effectively differentiated the MCI from CN groups (area under the curve (AUC = 0.74). Furthermore, the MBI-C effectively discriminated the iRBD patients with high phenoconversion risk against those with low-risk (cut-off 4.0, AUC = 0.72). The MBI-C may be a useful screening questionnaire reflecting clinical severity across the LBD continuum. Longitudinal studies are needed to determine its value in monitoring disease progression in prodromal LBD.

**Supplementary Information:**

The online version contains supplementary material available at 10.1007/s00702-024-02877-w.

## Introduction

A growing body of evidence suggests that emergent neuropsychiatric symptoms (NPS) in older adults serve as an early marker of cognitive decline and disease progression in various neurodegenerative diseases (Ballard et al. 2008; Monastero et al. 2009; Peters et al. 2013; Pink et al. 2015). The International Society to Advance Alzheimer’s Research and Treatment has introduced the concept of mild behavioral impairment (MBI) (Ismail et al. 2016). MBI is defined by the emergence of persistent (> 6 months) and impactful NPSs in non-demented individuals in later life, which is considered an at-risk state for incident cognitive impairment and dementia (Matuskova et al. 2021).

There are studies investigating the values of screening MBI for early detection of Alzheimer’s dementia (AD) or Lewy body disease (LBD)-related pathology in older adults. For AD continuum, presence of MBI is linked to future development of mild cognitive impairment (MCI) or dementia in cognitively normal (CN) individuals (Matsuoka et al. 2019; Ismail et al. 2021). MBI is also common in non-demented PD patients being associated with future disease progression, motor impairment, and cognitive deterioration (Baschi et al. 2019; Angelopoulou et al. 2024) However, it remains unrevealed as to potential diagnostic and prognostic values of MBI in individuals within LBD continuum, especially with prodromal LBD.

Previous studies have indicated that patients with idiopathic REM sleep behavior disorder (iRBD) commonly experience mild mood or psychiatric symptoms similar to those observed in PD (Barber et al. 2017). Notably, iRBD patients exhibited significantly heightened depression and anxiety compared to healthy controls and tended to demonstrate apathy even more frequently than PD patients (Barber et al. 2017). Identifying the progression of iRBD patients to clinically overt LBD is of substantial clinical importance, particularly considering the possible disease modifying interventions that are actively under investigation. (Postuma et al. 2019).

The MBI-checklist (MBI-C) was developed as a simple and efficient tool for MBI case ascertainment, primarily for being responded by patients or family members and close informants (Ismail et al. 2017). This feature makes the MBI-C particularly attractive for population-based studies, as it can help identify groups at higher risk of cognitive decline. Timely identification of high-risk individuals in the prodromal stage through a single questionnaire would be critically beneficial, as individuals in this stage often do not seek medical care due to the subtle nature of their symptoms.

There is a lack of comprehensive research on the possible role of MBI in detecting or monitoring disease progression in individuals within the LBD continuum, including the prodromal stage. Therefore, in the present study, we investigated the presence of MBI and its relationship with disease severity by stratifying participants according to their cognitive status. Furthermore, we also investigated whether MBI-C could aid in assessing the disease severity in prodromal LBD.

## Methods

### Study participants

We prospectively recruited a cohort with LBD continuum from the movement disorders clinic of Seoul National University Boramae Medical Center (SNU-BMC) between February 2022 and March 2023. The LBD continuum consisted of prodromal LBD with iRBD and established LBD with PD and dementia with Lewy bodies (DLB). The diagnosis of iRBD was based on the International Classification of Sleep Disorders, third Edition, and confirmed by video-polysomnography (video-PSG). PD and DLB diagnoses were based on the Movement Disorder Society Clinical Diagnostic Criteria, and the Fourth Consensus Report of the DLB Consortium (Postuma et al. 2015; McKeith et al. 2017), respectively. We excluded patients with secondary REM sleep behavior disorder due to psychiatric disorders or medications, as well as those with other neurological conditions besides PD, DLB or iRBD.

### Clinical evaluation

All participants were evaluated with the Movement Disorder Society-Unified Parkinson’s Disease Rating Scale (MDS-UPDRS), and the Korean version of the Scale for Outcomes in Parkinson’s Disease Autonomic Questionnaire (K-SCOPA-AUT) (Kim et al. 2017). We assessed MBI using the MBI-C (Ismail et al. 2017) questionnaire. The Korean version of the MBI-C is available at www.MBItest.org. The MBI-C was completed by informants, primarily family members of the patients, and in some cases, close caregivers. Cognition was assessed through the Seoul Neuropsychological Screening Battery, a comprehensive neuropsychological test battery, as previously described (Yoo et al. 2021). It comprised of the Digit Span Forward–Backward Test, Korean Color Word Stroop Test (K-CWST), Trail Making Test Parts A (TMT-A) and B (TMT-B), Controlled Oral Word Association Test (COWAT), Seoul Verbal Learning Test (SVLT), Rey Complex Figure Test (RCFT), Digit Symbol Coding (DSC), Korean version of the Boston Naming Test (K-BNT), Geriatric Depression Scale (GDS) and the Korean version of Mini-Mental State Examination (K-MMSE). The results were presented as z-scores adjusted for age and years of education, except for GDS and K-MMSE.

The iRBD cohort protocol of our center includes assessments such as olfactory function test, comprehensive neuropsychological test, K-SCOPA-AUT, MDS-UPDRS, video-PSG, and ^11^F-FP-CIT PET scan (Shin et al. 2020; Yoo et al. 2021; Woo et al. 2023, 2024). In this study, iRBD patients were evaluated for known prodromal markers of LBD, including olfaction, urinary dysfunction, orthostatic dizziness, constipation, mild parkinsonian sign (MPS), and MCI, in accordance with this protocol. MCI was defined according to the level II MDS criteria (Litvan et al. 2012). Olfactory function was evaluated using either the Butanol Threshold Test (BTT) or the Korean Version of Sniffin’ Sticks (KVSS) test (YOF test, Kimex Co., Suwon, Korea) (Woo et al. 2023). Hyposmia was defined as a BTT score below 6 or a total KVSS score below 22. MPS were defined as a motor score on the MDS-UPDRS > 6, excluding action and postural tremor (Buchanan et al. 2021). Constipation, urinary dysfunction, and orthostatic symptoms were assessed based on the MDS-UPDRS part 1. A score of 1 or higher was indicating the presence of these symptoms. We defined the iRBD-enriched risk group as subjects with three or more prodromal markers.

### Statistical analysis

Numerical variables were represented as means ± standard deviations or medians (interquartile range) following the verification of normal distribution with the Shapiro–Wilk test. Categorical variables were presented as numbers with percentages, and comparisons between categorical variables were performed using the chi-squared test or Fisher’s exact test. Two-sample t-tests or Mann–Whitney tests were applied for two-group comparisons of continuous variables, while one-way analysis of variance (ANOVA) with Tukey post-hoc tests or Kruskal–Wallis tests followed by Dunn’s test with Bonferroni correction were used for three-group comparisons. When comparisons between groups required adjustment for covariates such as age or years of education, analysis of covariance (ANCOVA) was performed. Following ANCOVA, post hoc analysis was conducted using pairwise Wilcoxon rank-sum tests with Bonferroni correction applied for multiple comparisons. Partial correlation analysis between clinical indices and MBI-C total scores was conducted, adjusting for age and sex as covariates. The Jonckheere-Terpstra test was used to assess trends in MBI across the LBD continuum. Sensitivity and specificity of MBI for discriminating between groups were assessed using receiver operating characteristic (ROC) curve analysis, along with Youden’s index to determine the optimal cutoff point for the MBI-C. All statistical analyses were conducted using R software (version 4.4.1; R Foundation for Statistical Computing, Vienna, Austria) with the significance level set at 0.05 (two-tailed).

## Results

### Characteristics of the participants

A total of 84 patients were included in this study, comprising 35 individuals with video PSG-confirmed iRBD, 29 with PD, and 20 with DLB patients. We categorized the participants into three groups according to the cognitive status; 19 LBD-Cognitively Normal (LBD-CN), 45 LBD-Mild Cognitive Impairment (LBD-MCI), and 20 Lewy body dementia. With the exception of age, there were no statistically significant differences among the three groups in terms of sex, years of education, or the duration of RBD, PD, or DLB. The detailed demographics and clinical features are presented in Table 1. Significant differences were observed among the three groups in MDS-UPDRS parts 1, 2, and 3, as well as in the total score. Moreover, these scores exhibited an increasing trend from CN to MCI, and then to dementia group (p < 0.001). Among the subdomains of the MDS-UPDRS part 1, we observed significant differences in hallucinations and psychosis (part 1.2, p = 0.001), depressed mood (part 1.3, p = 0.019), anxious mood (part 1.4, p = 0.001), and apathy (part 1.5, p = 0.014) scores across the three groups, even after adjusting for age. Additionally, there was an increasing trend in these subdomain scores from CN to MCI, and then to dementia groups. Neuropsychological tests demonstrated that the MCI and dementia group performed significantly worse than the CN group in attention (TMT-A; p = 0.004), language (K-BNT; *p* < 0.001), memory (RCFT delayed recall; p = 0.017), and frontal/executive functions (COWAT phonemic; *p* < 0.001, K-CWST color reading; *p* < 0.001, DSC; *p* = 0.012, TMT-B; *p* = 0.007).

**Table 1:** Characteristic of the subject with Lewy body disease (LBD) continuum

	Normal cognition (n = 19)	Mild cognitive impairment (n = 45)	Dementia (n = 20)	*p* value
Age, y	70.2 (6.6)	70.4 (6.9)	75.1 (6.4)	**0.027**^a^
Sex (F/M)	10/9	19/26	10/10	0.699^b^
iRBD/PD	12/7	23/22	N/A	** < 0.001**^b^
Education, y	12.0 (8.5 ~ 14.0)	9.00 (6.0 ~ 12.0)	6.0 (6.0 ~ 10.5)	0.050^c^
K-MMSE	28.00 (26.00 ~ 30.00)	26.00 (23.00 ~ 28.00)	23.50 (17.75 ~ 25.25)	** < 0.001**
GDS	9.00 (6.00 ~ 17.0)	15.00 (9.00 ~ 23.50)	13.50 (8.75 ~ 24.00)	0.162
K-SCOPA-AUT total	10.00 (6.50 ~ 11.00)	10.00 (7.00 ~ 19.00)	17.00 (11.50 ~ 22.00)	0.134
MDS-UPDRS-I	7.00 (4.50 ~ 11.50)	9.00 (7.00 ~ 15.00)	16.50 (10.00 ~ 18.50)	**0.002**
MDS-UPDRS-II	2.00 (0.50 ~ 7.00)	5.00 (1.00 ~ 12.00)	10.50 (8.00 ~ 18.25)	**0.001**
MDS-UPDRS-III	11.00 (5.00 ~ 14.25)	13.00 (4.00 ~ 26.50)	34.50 (22.00 ~ 43.00)	** < 0.001**
MDS-UPDRS-total	22.00 (12.25 ~ 34.00)	27.00 (17.00 ~ 45.50)	57.25 (41.50 ~ 78.88)	** < 0.001**
Neuropsychological battery^a,c^				
DST F-B	0.02 (−0.85 ~ 0.80)	−0.02 (−0.67 ~ 0.67)	−0.01 (−0.83 ~ 0.69)	0.894
TMT-A	0.76 (0.26 ~ 1.06)	0.29 (−0.59 ~ 0.79)	−0.55 (−0.99 ~ 0.23))	**0.004**
K-BNT	0.50 (0.75)	−0.41 (1.16)	−0.90 (1.01)	** < 0.001**
RCFT copy score	−0.47 (−1.80 ~ −0.23)	−1.66 (−2.43 ~ −1.09)	−1.52 (−2.23 ~ −1.07)	0.060
SVLT immediate recall	0.39 (1.14)	−0.59 (1.26)	−0.92 (0.85)	**0.003**
SVLT delayed recall	−0.01 (1.19)	−0.68 (1.37)	−0.96 (0.87)	0.070
SVLT recognition	0.15 (0.81)	−0.38 (1.10)	−0.59 (0.85)	0.076
RCFT immediate recall	−0.16 (0.90)	−0.62 (0.89)	−1.00 (0.73)	**0.021**
RCFT delayed recall	0.03 (−0.79 ~ 0.36)	−0.56 (−1.22 ~ −0.05)	−1.24 (−1.49 ~ −0.65)	**0.017**
RCFT recognition	0.15 (−0.22 ~ 0.80)	−0.28 (−1.22 ~ 0.09)	−0.28 (−1.22 ~ −0.05)	**0.005**
COWAT phonemic	0.24 (0.90)	−0.76 (0.89)	−0.65 (0.80)	** < 0.001**
COWAT semantic	0.16 (0.78)	−0.67 (0.70)	−0.54 (0.71)	** < 0.001**
K-CWST color reading	0.43 (0.06 ~ 0.66)	−0.57 (−1.16 ~ 0.31)	−1.36 (−1.93 ~ −0.86)	** < 0.001**
DSC	0.60 (1.02)	−0.20 (1.25)	−0.54 (1.05)	**0.012**
TMT-B	0.22 (−2.46 ~ 0.96)	−2.07 (−5.52 ~ 0.28)	−2.90 (−5.95 ~ −2.11)	**0.007**

*COWAT* Controlled Oral Word Association Test; *DSC* Digit Symbol Coding; *DST F-B* Digit Span Test forward–backward; GDS Geriatric depression scale; *iRBD* isolated REM sleep behavior disorder; *K-BNT* Korean version of Boston Naming Test; *K-CWST* Korean Color Word Stroop Test; *K-MMSE* = Korean version of Mini-Mental State Examination; *K-SCOPA-AUT* Korean version of the Scale for Outcomes in Parkinson’s disease-Autonomic; *MDS-UPDRS* Movement Disorder Society-sponsored revision of the Unified Parkinson’s Disease Rating Scale; *PD* Parkinson’s Disease; *RCFT* Rey-Osterrieth Complex Figure Test; *SVLT* Seoul Verbal Learning Test; *TMT* Trail-Making Test

Datas are presented mean (standard deviation) or median (interquartile range). Bold values indicate statistically significant results with *p* values less than 0.05

Analysis of covariance was performed using age and years of education as a covariate, except where indicated otherwise.

^a^One-way ANOVA with post-hoc Tukey’s HSD test

^b^Pearson’s chi-squared test

^c^Kruskal-Wallis test with post-hoc Dunn’s test

### MBI across the LBD continuum

**Fig. 1:**
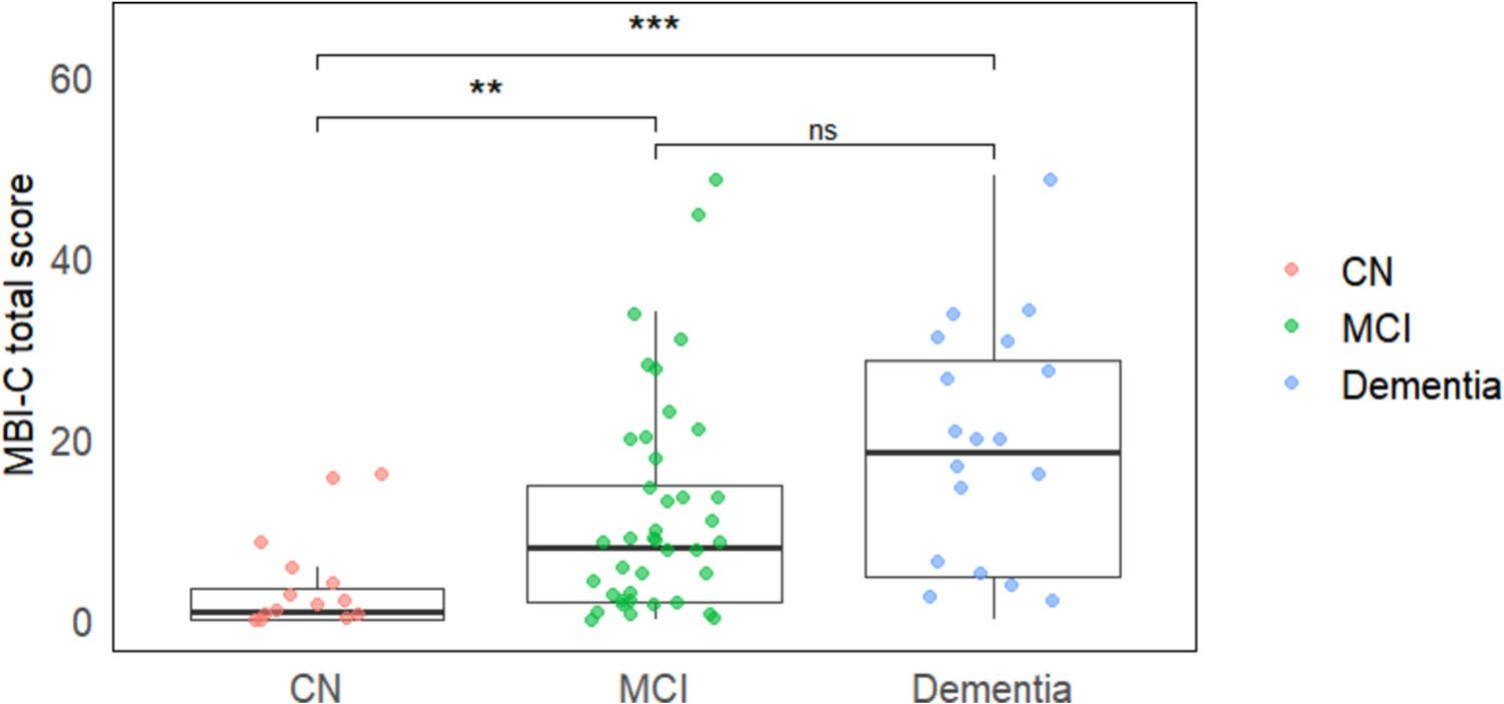
MBI-C total scores of LBD continuum. The MBI-C total score showed a statistically significant increasing trend across the three groups, with significant differences between CN and MCI groups, and between CN and dementia groups (p < 0.001). ** p < 0.01, *** p < 0.001, *ns*; not significant

The MBI-C total score showed significant differences among the three groups, with an increasing trend from CN to MCI, and then to dementia groups (p < 0.001) (Table 2, Fig. 1). In the MBI-C subdomains, significant group differences were observed, with the most pronounced differences noted in the subdomains of decreased motivation, affective dysregulation, and impulse dyscontrol.

**Table 2:** Comparison of MBI-C total and subdomain scores across the LBD continuum

	Normal cognition (n = 19)	Mild cognitive impairment (n = 45)	Dementia (n = 20)	*p* value
MBI-C total	1.00 (0.00 ~ 3.50)	8.00 (2.00 ~ 15.00)	18.50 (4.75 ~ 28.75)	** < 0.001**^a,b^
MBI-C subdomains				
Decreased motivation	0.00 (0.00 ~ 1.00)	1.00 (0.00 ~ 3.00)	5.00 (0.00 ~ 8.50)	**0.006**^b^
Affective dysregulation	0.00 (0.00 ~ 2.00)	2.00 (0.00 ~ 5.00)	4.00 (1.75 ~ 6.25)	**0.026**^a,b^
Impulse dyscontrol	0.00 (0.00 ~ 1.00)	2.00 (0.00 ~ 6.00)	2.50 (1.75 ~ 8.00)	**0.007**^a,b^
Social inappropriateness	0.00 (0.00 ~ 0.00)	0.00 (0.00 ~ 1.00)	1.50 (0.00 ~ 4.00)	**0.006**^b^
Abnormal thought and perception	0.00 (0.00 ~ 0.00)	0.00 (0.00 ~ 1.00)	0.50 (0.00 ~ 4.00)	**0.012**^b^

*MBI-C* Mild Behavioral Impairment-Checklist

Analysis of covariance was performed using age and years of education as a covariate, followed by post hoc pair-wise Wilcoxon rank sum tests. Bold values indicate statistically significant results with *p* values less than 0.05

^a^Significant difference between CN and MCI

^b^Significant difference between CN and dementia

^c^Significant difference between MCI and dementia

We performed partial correlation analyses in the entire LBD continuum subjects, controlling for age and sex to assess the relationship between the MBI-C total score and various clinical indices (Table 3). Significant associations were found with GDS, K-MMSE, and the MDS-UPDRS parts 1, 2, 3, and total scores. Regarding neuropsychological tests, significant correlations were observed in domains such as attention (TMT-A; r = −0.343, p = 0.002), language (K-BNT; r = −0.354, p = 0.001), memory (SVLT immediate recall; r = −0.250, p = 0.026), and frontal/executive functions (DSC; r = −0.314, p = 0.006, TMT-B; r = −0.339, p = 0.003). When partial correlation analysis was applied to subgroups within the LBD continuum, slight variations in the patterns of associations across groups were noted. In the LBD-CN group, significant correlations were observed with GDS (r = 0.505, p = 0.039), and the frontal/executive function domains, specifically COWAT phonemic (r = −0.482, p = 0.036) and COWAT semantic (r = −0.477, p = 0.039). The LBD-MCI group showed significant associations with the MDS-UPDRS parts 1, 2, 3, and total scores, as well as with attention (TMT-A; r = −0.311, p = 0.040) and frontal/executive functions (DSC; r = −0.329, p = 0.033, TMT-B; r = −0.303, p = 0.048). In the Lewy body dementia group, correlations were found with the MDS-UPDRS total score (r = 0.568, p = 0.022), global cognition as measured by the K-MMSE (r = −0.582, p = 0.018), and verbal memory function (SVLT delayed recall; r = −0.719, p = 0.003).

**Table 3:** Association of MBI-C total score with clinical indices across the entire LBD continuum and its subpopulations

	Total (n = 84)		Normal cognition (n = 19)		Mild cognitive impairment (n = 45)		Dementia (n = 20)	
	r	*p* value	r	*p* value	r	*p* value	r	*p* value
MDS-UPDRS-I	**0.504**	** < 0.001**	0.349	0.143	**0.473**	**0.001**	0.447	0.083
MDS-UPDRS-II	**0.418**	** < 0.001**	0.175	0.473	**0.433**	**0.004**	0.497	0.050
MDS-UPDRS-III	**0.458**	** < 0.001**	−0.093	0.705	**0.373**	**0.014**	0.459	0.074
MDS-UPDRS total	**0.508**	** < 0.001**	0.163	0.505	**0.477**	**0.001**	**0.568**	**0.022**
GDS	**0.459**	** < 0.001**	**0.505**	**0.039**	**0.439**	**0.003**	**0.603**	**0.013**
K-MMSE	**−0.430**	** < 0.001**	−0.033	0.892	−0.155	0.321	−**0.582**	**0.018**
Neuropsychological battery								
DST F-B	−0.150	0.191	0.336	0.160	−0.165	0.286	−**0.620**	**0.014**
TMT-A	−**0.343**	**0.002**	**−**0.279	0.247	**−0.311**	**0.040**	−0.002	0.995
K-BNT	−**0.364**	**0.001**	**−**0.122	0.620	**−**0.242	0.110	−0.365	0.181
RCFT copy score	−0.090	0.428	0.120	0.624	**−**0.036	0.812	0.059	0.834
SVLT immediate recall	−**0.250**	**0.026**	0.323	0.178	**−**0.106	0.490	−0.453	0.090
SVLT delayed recall	−**0.230**	**0.041**	0.106	0.667	**−**0.111	0.469	−**0.719**	**0.003**
SVLT recognition	−0.163	0.152	0.046	0.853	**−**0.158	0.301	0.082	0.770
RCFT immediate recall	−0.176	0.122	0.100	0.685	**−**0.251	0.096	0.321	0.243
RCFT delayed recall	−0.185	0.103	0.118	0.631	**−**0.279	0.064	0.259	0.351
RCFT recognition	−**0.229**	**0.043**	**−**0.082	0.738	**−**0.026	0.864	−**0.524**	**0.045**
COWAT phonemic	−**0.247**	**0.029**	**−0.482**	**0.036**	**−**0.134	0.387	−0.125	0.656
COWAT semantic	−**0.229**	**0.042**	**−0.477**	**0.039**	**−**0.108	0.478	−0.021	0.939
K-CWST color reading	−0.265	0.051	**−**0.133	0.625	0.062	0.746	**−**0.217	0.581
DSC	−**0.314**	**0.006**	0.063	0.798	**−0.329**	**0.033**	**−**0.172	0.540
TMT-B	**−0.338**	**0.003**	**−**0.163	0.506	**−0.303**	**0.048**	**−**0.042	0.881

*COWAT* Controlled Oral Word Association Test; *DSC* Digit Symbol Coding; *DST F-B* Digit Span Test forward–backward; *GDS* Geriatric depression scale; *K-BNT* Korean version of Boston Naming Test; *K-CWST* Korean Color Word Stroop Test; *K-MMSE* Korean version of Mini-Mental State Examination; MDS-*UPDRS* Movement Disorder Society-sponsored revision of the Unified Parkinson’s Disease Rating Scale; *RCFT* Rey-Osterrieth Complex Figure Test; *SVLT*  Seoul Verbal Learning Test; *TMT* Trail-Making Test

Spearman’s rank partial correlation analysis was performed, adjusting for age and sex, except where indicated otherwise. Bold values indicate statistically significant results with *p* values less than 0.05

For differentiating MCI from CN in the LBD continuum, the area under the curve (AUC) of the MBI-C was found to be 0.736 [95% confidence interval (CI) = 0.607–0.965, p = 0.001], with an optimal cutoff point of 5.0, sensitivity of 60.0%, and specificity of 78.9%.

### MBI as a risk marker in iRBD

The iRBD group was categorized into two subgroups based on the presence or absence of multiple prodromal markers. Out of the 35 total iRBD patients, 11 were classified into the iRBD-low risk group, while 24 were categorized into the iRBD-enriched risk group. Detailed demographics and clinical features are presented in Supplementary Table 1. All six prodromal markers were more prevalent in the iRBD-enriched risk compared to low risk groups, with significant differences in MPS (p = 0.003), constipation (p = 0.002), and orthostatic dizziness (p = 0.038) (data not shown). The MBI-C total score was higher in the iRBD-enriched risk group. The MBI-C’s five subdomains showed notable distinctions, particularly in the domain of impulse dyscontrol (*p* = 0.003). The MDS-UPDRS parts 1, 2, 3, and total scores were all significantly higher in the iRBD-enriched risk group, a pattern that was also observed in the K-SCOPA-AUT total score. In neuropsychological tests, the iRBD-enriched risk group exhibited a non-significant tendency of lower performance in the frontal/executive function than the iRBD-low risk group (supplementary Table 1). There was no significant relationship between the RBD symptom duration and MBI in iRBD patients (data not shown).

For discriminating the iRBD-enriched risk against the low-risk groups, ROC analysis revealed that the AUC of the MBI-C was 0.720 [95% CI 0.559–0.881,* p* = 0.018]. The optimal cut-off point of the MBI-C was 4.0, with a sensitivity of 58.3% and a specificity of 90.9%.

## Discussion

This study is the first to investigate the potential utility of the MBI-C in the LBD continuum, including the prodromal stage. We demonstrated the moderate diagnostic values of MBI-C in screening individuals with MCI-LB from CN states and iRBD-enriched risk from the low-risk subjects, thereby suggesting the usefulness of this questionnaire in screening individuals with disease progression or high-risk phenoconversion within the LBD continuum. Although the MBI-C total score cannot serve as a gold standard test for diagnosing or assessing disease progression, it may hold clinical utility arisen from its advantage of easy screening by patients or their caregivers.

This study employed nearly 42% of the cohort with iRBD patients at the prodromal stage and revealed significant correlations between the MBI-C and motor and nonmotor MDS-UPDRS scores and prodromal cognitive functions. This underscores the broader applicability of the MBI-C across the entire LBD continuum, compared to previous studies that focused primarily on PD patients (Baschi et al. 2019; Yoon et al. 2019; Lang et al. 2020; Ramezani et al. 2021; Monchi et al. 2024).

Recent research has distinguished the presence of MCI in PD patients using an MBI-C cutoff of 5.0 (Monchi et al. 2024), which was similar the cutoffs determined in the present study. Whether in the context of established motor LBD such as PD or within a broader LBD continuum, similar MBI-C cutoff points have proven effective across various populations. This indicates that the MBI-C has the potential for broader application in distinguishing different stages and phenotypes of LBD.

The MBI-C is a questionnaire that can be screened at home, so its ease of use could facilitate the recruitment of such prodromal patients to clinic visits. If these patients undergo regular follow-up, it will enable the timely detection of phenoconversion without missing any critical time points. Effective screening is crucial not only for those in the prodromal LBD stage but also for the LBD-MCI group. A higher MBI-C total score identifies subpopulations with a greater likelihood of progression to Lewy body dementia, such as PD dementia (PDD) or DLB. Therefore, implementing comprehensive cognitive assessments, in conjunction with the straightforward and time-efficient MBI-C, allows for smoother documentation of cognitive progression starting from the cognitively normal stage. While the MBI-C will primarily serve as a screening tool, it is poised to play a pivotal role in identifying at-risk subpopulations within the prodromal stage that may be overlooked in clinical practice. By facilitating their referral to clinical visits, it ensures these individuals receive timely and objective assessments by specialists. This can ultimately enable appropriate therapeutic interventions at a critical juncture.

Our study has a notable strength. As previously mentioned, there has been no prospective cohort study encompassing comprehensive motor and non-motor symptoms in LBD patients, including those with iRBD. The iRBD participants in this study were exclusively composed of polysomnography-confirmed patients, ensuring that we have properly gathered individuals in the prodromal LBD stage.

Some limitations of our work should be acknowledged. First, we did not consider prodromal Multiple System Atrophy (MSA) in our iRBD population, as it is known that only 6% of individuals with iRBD may phenoconvert to MSA, which explains maximum 2 patients may have a potential of MSA in our study participants. The sample size of this study is relatively small, with only 35 iRBD patients, which limits the generalizability of our findings. Additionally, the cross-sectional nature of this study presents another significant limitation, as it does not allow for an assessment of the temporal relationship between the presence of prodromal markers and the progression to clinically established LBD. To more rigorously validate the utility of the MBI-C as screening tool for phenoconversion risk within the LBD continuum, future studies with larger sample size, longer follow-up periods, and employing the MBI-C in combination with established clinical evaluations and biomarkers (e.g., biofluid and imaging markers) will be necessary (Miglis et al. 2021). Furthermore, it would be beneficial for future research to include patients with PDD in the Lewy body dementia group to provide a more comprehensive analysis and enhance the understanding of disease patterns within this subgroup.

In conclusion, the MBI may serve as a potential marker of high-risk phenoconversion and motor and non-motor clinical severity across the LBD continuum. The MBI-C questionnaire may be a useful screening method with easy accessibility allowing the scoring performed without direct clinician involvement. Further longitudinal studies are needed to determine whether the MBI-C can be effectively used to monitor disease progression in the LBD continuum population.

## Supplementary Information

Below is the link to the electronic supplementary material.

Supplementary file1 (DOCX 23 KB)

## Data Availability

The data that support the findings of this study are available from the corresponding author upon reasonable request.
