# Supplementary material for: Mild behavioral impairment in idiopathic REM sleep behavior disorder and Lewy body disease continuum
Source: J Neural Transm (Vienna). 2025 Jan 9;132(5):637–44. doi: 10.1007/s00702-024-02877-w (PMC12043749; doi:10.1007/s00702-024-02877-w)
Supplement: Supplementary file 1 — Supplementary file1 (DOCX 23 KB) [file 702_2024_2877_MOESM1_ESM.docx]

**Supplementary table 1.** Comparisons of characteristics between iRBD groups

|  | iRBD-low risk  (n=11) | iRBD-enriched risk  (n=24) | *p* value |
| --- | --- | --- | --- |
| Age, y | 67.36 (7.07) | 72.50 (4.91) | **0.018**^a^ |
| Sex (F/M) | 7/4 | 10/14 | 0.399^b^ |
| Education, y | 9.00 (7.00~14.00) | 7.50 (5.75~12.50) | 0.315^c^ |
| MBI-C total | 1.00 (0.00~2.00) | 5.50 (0.00~14.00) | **0.024** |
| Decreased motivation | 0.00 (0.00~0.00) | 1.00 (0.00~3.00) | **0.033** |
| Affective dysregulation | 0.00 (0.00~1.00) | 2.00 (0.00~4.25) | **0.042** |
| Impulse dyscontrol | 0.00 (0.00~0.00) | 1.00 (0.00~4.50) | **0.003** |
| Social inappropriateness | 0.00 (0.00~0.00) | 0.00 (0.00~1.00) | **0.004** |
| Abnormal thought and perception | 0.00 (0.00~0.00) | 0.00 (0.00~0.00) | 0.837 |
| MDS-UPDRS-I | 4.00 (3.50~5.50) | 9.00 (7.00~13.00) | **0.001** |
| MDS-UPDRS-II | 0.00 (0.00~1.00) | 3.50 (1.00~7.00) | **0.001** |
| MDS-UPDRS-III | 1.00 (0.00~3.50) | 8.25 (4.00~11.50) | **<0.001** |
| MDS-UPDRS total | 7.50 (3.45) | 24.02 (10.50) | **<0.001** |
| K-SCOPA-AUT total | 6.00 (3.00~6.00) | 10.00 (7.75~13.25) | **0.001** |
| K-MMSE | 27.00 (26.00~28.50) | 28.00 (24.50~29.00) | 0.971 |
| GDS | 8.00 (6.50~10.00) | 9.00 (6.00~19.00) | 0.292 |
| Neuropsychological battery^a,c^ |  |  |  |
| DST F-B | -0.07 (0.70) | 0.12 (0.88) | 0.551 |
| TMT-A | 0.79 (0.09~1.00) | 0.60 (-0.17~0.89) | 0.407 |
| K-BNT | 0.40 (0.82) | -0.11 (1.24) | 0.222 |
| RCFT copy score | -1.10 (1.04) | -1.28 (1.41) | 0.708 |
| SVLT immediate recall | -0.16 (1.37) | -0.11 (1.32) | 0.926 |
| SVLT delayed recall | -0.55 (1.21) | -0.36 (1.15) | 0.671 |
| SVLT recognition | -0.20 (0.95) | 0.00 (1.02) | 0.573 |
| RCFT immediate recall | -0.36 (1.16) | -0.37 (0.87) | 0.972 |
| RCFT delayed recall | -0.28 (0.81) | -0.32 (0.83) | 0.904 |
| RCFT recognition | -0.23 (0.66) | -0.04 (1.03) | 0.588 |
| COWAT phonemic | 0.06 (1.12) | -0.47 (0.91) | 0.153 |
| COWAT semantic | 0.05 (0.92) | -0.42 (0.73) | 0.115 |
| K-CWST color reading | -0.07 (1.11) | -0.09 (1.18) | 0.962 |
| DSC | 0.59 (0.77) | 0.02 (0.77) | 0.055 |
| TMT-B | 0.39 (-3.51~0.83) | -0.79 (-3.75~0.28) | 0.473 |

Abbreviations: COWAT=Controlled Oral Word Association Test; DSC=Digit Symbol Coding; DST F-B=Digit Span Test forward-backward; GDS=Geriatric depression scale; iRBD=isolated REM sleep behavior disorder; K-BNT=Korean version of Boston Naming Test; K-CWST=Korean Color Word Stroop Test; K-MMSE= Korean version of Mini-Mental State Examination; K-SCOPA-AUT=Scale for Outcomes in Parkinson’s disease-Autonomic; MBI-C=Mild Behavioral Impairment-Checklist; MDS-UPDRS=Movement Disorder Society-sponsored revision of the Unified Parkinson’s Disease Rating Scale; RCFT=Rey-Osterrieth Complex Figure Test; SVLT=Seoul Verbal Learning Test; TMT=Trail-Making Test

Datas are presented mean (standard deviation) or median (interquartile range).

Analysis of covariance was performed using age and years of education as a covariate, except where indicated otherwise.

^a^Independent t test.

^b^Pearson’s chi-squared test.

^c^Mann-Whitney U test.
